# Measuring Ostracism-Induced Changes in Consumption of Palatable Food: Feasibility of a Novel Behavioral Task

**DOI:** 10.3389/fpsyg.2022.853555

**Published:** 2022-05-17

**Authors:** Kristin N. Javaras, Erin M. LaFlamme, Lauren L. Porter, Meghan E. Reilly, Chris Perriello, Harrison G. Pope, James I. Hudson, Staci A. Gruber, Shelly F. Greenfield

**Affiliations:** ^1^Division of Women’s Mental Health, McLean Hospital, Belmont, MA, United States; ^2^Department of Psychiatry, Harvard Medical School, Boston, MA, United States; ^3^Biological Psychiatry Laboratory, McLean Hospital, Belmont, MA, United States; ^4^Division of Alcohol, Drugs, and Addiction, McLean Hospital, Belmont, MA, United States; ^5^Cognitive and Clinical Neuroimaging Core, McLean Hospital, Belmont, MA, United States

**Keywords:** stressor, interpersonal relations, ostracism, rejection, emotion, eating behavior, palatable food, eating disorder

## Abstract

**Purpose:**

Ostracism is a highly aversive interpersonal experience. Previous research suggests that it can increase consumption of highly palatable food in some individuals, but decrease it in others. Thus, we developed the Cyberball-Milkshake Task (CMT), to facilitate research investigating individual differences in ostracism’s effects on consumption of highly palatable food. We present data on feasibility for the CMT in a sample of young adult women.

**Materials and Methods:**

Participants were 22 women, 18–30 years old, reporting very low or very high levels of emotional eating at screening. Participants performed the CMT, which consisted of 12 trials. Each trial included: playing a round of Cyberball (a computerized game of catch with fictitious “other participants” programmed to either include or exclude the participant); viewing a chocolate image; and then consuming a participant-determined amount of milkshake. Participants subsequently played an additional inclusion and exclusion round of Cyberball, each immediately followed by questionnaires assessing current mood and recent Cyberball experience.

**Results:**

Cyberball exclusion (vs. inclusion) was associated with large, significant increases in reported ostracism and threats to self-esteem; exclusion’s effects on affect were in the expected direction (e.g., increased negative affect), but generally small and non-significant. Milkshake intake was measurable for 95% of participants, on 96% of trials. Intake decreased quadratically across trials, with a steep negative slope for low trial numbers that decreased to the point of being flat for the highest trial numbers.

**Discussion:**

The CMT is a generally feasible approach to investigating ostracism’s effects on consumption of highly palatable food. The feasibility (and validity) of the CMT may benefit from modification (e.g., fewer trials and longer rounds of Cyberball). Future research should examine whether performance on a modified version of the CMT predicts real-world behavior in a larger sample.

## Introduction

Ostracism and rejection are highly aversive interpersonal experiences that cause pain and distress (Williams, 2007), at least in Western cultures (Uskul and Over, 2017). These experiences can also threaten fundamental intrapersonal needs, such as feelings of belonging and self-esteem (Hartgerink et al., 2015), and can result in increased negative (Williams, 2007) and decreased positive (Lustenberger and Jagacinski, 2010) affect. Ostracism and rejection can also elicit a wide range of behavioral responses ranging from prosocial to antisocial. Notably, although these behaviors may serve to fulfill needs threatened by ostracism, they are not necessarily adaptive for the ostracized individual (Williams, 2007).

With respect to eating behavior, several experimental studies have examined whether ostracism affects consumption of highly palatable food in general adult samples. In two mixed-gender samples, ostracism or rejection had a main effect on cookie consumption, with ostracized or rejected participants consuming two to three times as many cookies immediately after the experience, compared to included or accepted participants (Baumeister et al., 2005; Oaten et al., 2008). In contrast, three other studies found that ostracism led to increased consumption of highly palatable food only for certain individuals, or only in certain conditions. In a mixed-gender sample, participants reporting habitual stress hyperphagia (specifically, a tendency to eat more in response to stress caused by others) ate more ice cream when rejected than accepted, whereas participants reporting habitual stress hypophagia exhibited the opposite pattern of ice cream consumption (Sproesser et al., 2014). Similarly, in a study of women, participants scoring high on self-reported restrained eating ate more after rejection than after achievement-oriented stressors and a control condition, but the same was not true for women low on restraint (Tanofsky-Kraff et al., 2000). In a study of African-American women, ostracized (vs. included) participants ate more potato chips (although not chocolate candy), and only when excluded by white women and not when excluded by other African-American women (Hayman et al., 2015). Collectively, these studies suggest that some individuals consume more highly palatable food in response to ostracism, but that the effects of ostracism on eating behavior likely differ across individuals.

In order to facilitate research investigating individual differences in ostracism’s effect on eating behavior, we developed the Cyberball-Milkshake Task (CMT) to examine the effects of ostracism on consumption of a highly palatable food, namely, chocolate milkshake. In the CMT, ostracism is induced *via* Cyberball, a computerized game of catch with fictitious other participants who are programmed to either include or exclude the participant, with exclusion during Cyberball shown to consistently induce social distress (Williams and Jarvis, 2006). We aimed to develop a task that, with some adaptation for the neuroimaging context, could be used to investigate the neural underpinnings of ostracism’s effects on eating behavior (Sebastian et al., 2011). The primary aim of the present study was to examine task feasibility in a sample of individuals reporting varying eating patterns. In particular, we aimed to test whether brief, repeated rounds of exclusion during Cyberball would be sufficient to evoke feelings of ostracism, threaten intrapersonal needs, and increase negative and decrease positive affect. We also sought to assess whether milkshake intake could be measured on a trial-by-trial basis. Our secondary aim was to preliminarily examine how ostracism affected milkshake intake in the sample overall, and among individuals reporting varying levels of emotional eating on the Dutch Eating Behavior Questionnaire (DEBQ; van Strien et al., 1986). Because Cyberball ostracism has not consistently increased food intake (Oaten et al., 2008; Hayman et al., 2015), we did not have an *a priori* hypothesis regarding whether ostracism would increase milkshake intake in the sample as a whole. However, given evidence that Cyberball can increase negative affect (Williams, 2007), together with evidence that high self-reported emotional eating can predict greater food intake following a negative mood induction (van Strien, 2010), we hypothesized that ostracism would increase intake in individuals reporting higher levels of emotional eating.

## Materials and Methods

### Participants

Participants were recruited from the community in response to advertisements seeking young women (including those experiencing binge-eating) for a study on “cognitive processing and behavior,” posted on Rally,^1^ and at area colleges/universities, professional and vocational training schools, and retail locations.

Participants (see Table 1) were 18- to 30-year-old adults identifying primarily as female in gender, a group at increased risk of eating disorders (Hudson et al., 2007; Javaras and Hudson, 2015). To increase the likelihood that participants would demonstrate a range of changes in milkshake intake in response to Cyberball exclusion, we selected participants with either very low or very high responses (average response <1.6, or > 3.6, respectively) to a subset of modified items from the Emotional Eating subscale of the DEBQ (Bohon et al., 2009) at screening. We did not exclude individuals with eating disorders (other than anorexia nervosa) or with most other psychiatric disorders. Additional eligibility criteria (see Supplementary Material Section 1) were intended to ensure safety and to reduce the likelihood that participants would respond atypically to Cyberball, would consume too little milkshake to be measurable, would be aware of the CMT measurement aim, or would be unable to complete the study protocol. For example, individuals with anorexia nervosa were excluded because avoidance of high calorie foods, especially those high in fat (Mayer et al., 2012), could result in little to no milkshake being consumed.

**Table 1:** Demographic and other information for participants.^a^

	Low self-reported emotional eating^b^ (*n* = 9)			High self-reported emotional eating^b^ (*n* = 11)		
Age	23.2	(4.0)	[18, 28]	23.1	(3.8)	[18, 30]
Race						
American Indian or Alaska Native	0	(0.0%)		0	(0.0%)	
Native Hawaiian or Other Pacific Islander	0	(0.0%)		0	(0.0%)	
Asian	1	(11.1%)		0	(0.0%)	
Black or African-American	0	(0.0%)		0	(0.0%)	
White	7	(77.8%)		10	(90.9%)	
Other	1	(11.1%)		1	(9.1%)	
Ethnicity						
Hispanic or Latina	1	(11.1%)		1	(9.1%)	
Non-Hispanic or Latina	8	(88.9%)		10	(90.9%)	
Body mass index^c^	24.7	(5.0)	[20.2, 32.4]	24.5	(5.0)	[19.1, 36.0]
DEBQ Emotional Eating	1.5	(0.6)	[1.0, 2.9]	3.4	(0.7)	[1.9, 4.2]
Past psychiatric diagnoses^d^						
Major depressive disorder	0	(0.0%)		5	(45.5%)	
Panic disorder	0	(0.0%)		0	(0.0%)	
Anorexia nervosa	0	(0.0%)		2	(18.2%)	
Restricting	0	(0.0%)		1	(9.1%)	
Binge-eating/purging	0	(0.0%)		1	(9.1%)	
Bulimia nervosa	1	(11.1%)		0	(0.0%)	
Binge-eating disorder	1	(11.1%)		3	(27.3%)	
Current psychiatric diagnoses^d^						
Attention-deficit/hyperactivity disorder	0	(0.0%)		1	(9.1%)	
Combined	0	(0.0%)		0	(0.0%)	
Predominantly inattentive	0	(0.0%)		1	(9.1%)	
Predominantly hyperactive/impulsive	0	(0.0%)		0	(0.0%)	
Major depressive disorder	0	(0.0%)		1	(9.1%)	
Social anxiety disorder	1	(11.1%)		1	(9.1%)	
Panic disorder	0	(0.0%)		0	(0.0%)	
Agoraphobia	0	(0.0%)		0	(0.0%)	
Generalized anxiety disorder	0	(0.0%)		0	(0.0%)	
Obsessive–compulsive disorder	0	(0.0%)		0	(0.0%)	
Posttraumatic stress disorder	0	(0.0%)		0	(0.0%)	
Bulimia nervosa	0	(0.0%)		1	(9.1%)	
Binge-eating disorder	0	(0.0%)		2	(18.2%)	
Alcohol use disorder (12 mo.)	1	(11.1%)		4	(36.4%)	
Non-alcohol substance use disorder (12 mo.)	0	(0.0%)		1	(9.1%)	

DEBQ, Dutch Eating Behavior Questionnaire.

a For continuous variables, statistics include mean (standard deviation) [range]. For categorical variables, statistics include number (percentage) for each category.

b Participants had low or high average responses to a subset of modified items from the DEBQ Emotional Eating scale at screening.

c Two individuals with low self-reported emotional eating were missing body mass index values due to equipment problems.

d Disorders that were exclusionary (e.g., lifetime psychotic disorder and current/recent anorexia nervosa) are not included in table.

Of 22 eligible participants, one did not complete the task (for reasons unrelated to the study), and one participant was removed from analysis after preliminary inspection revealed highly implausible values calculated for milkshake intake (e.g., a calculated value of −300 g), which suggested measurement error.

### Procedures

Participants were asked not to consume alcohol, nicotine, recreational drugs, or excessive caffeine on the day before or the day of the study visit, and to eat a mid-day meal (e.g., turkey sandwich and piece of fruit) between 12-1 pm on the day of the visit. The 3.5-h visit included: milkshake tasting, to ensure that participants liked the milkshake; several semi-structured interviews assessing medical and psychiatric history; several “initial” questionnaires; an approximately 15 min break; a 15 min baseline period, during which participants listened to music (John Adams’ *Common Tones in Simple Time*) previously used in neutral mood inductions (Bohon et al., 2009); the CMT, which occurred at approximately 3:30 pm, followed by completion of several “task-related” questionnaires; part one of a funnel debriefing interview; rating of chocolate images used in the CMT; “final” questionnaires, which included the questionnaires most likely to cue participants to the CMT’s measurement aim; anthropometric measurement, including height and weight; part two of the funnel debriefing interview; and debriefing and safety assessment.

Participants received monetary compensation for adherence to the pre-visit instructions and for the study visit. Participants provided informed consent before beginning any study procedures, and the study was approved by the MassGeneral Brigham Institutional Review Board.

### Cyberball-Milkshake Task

**Figure 1:**
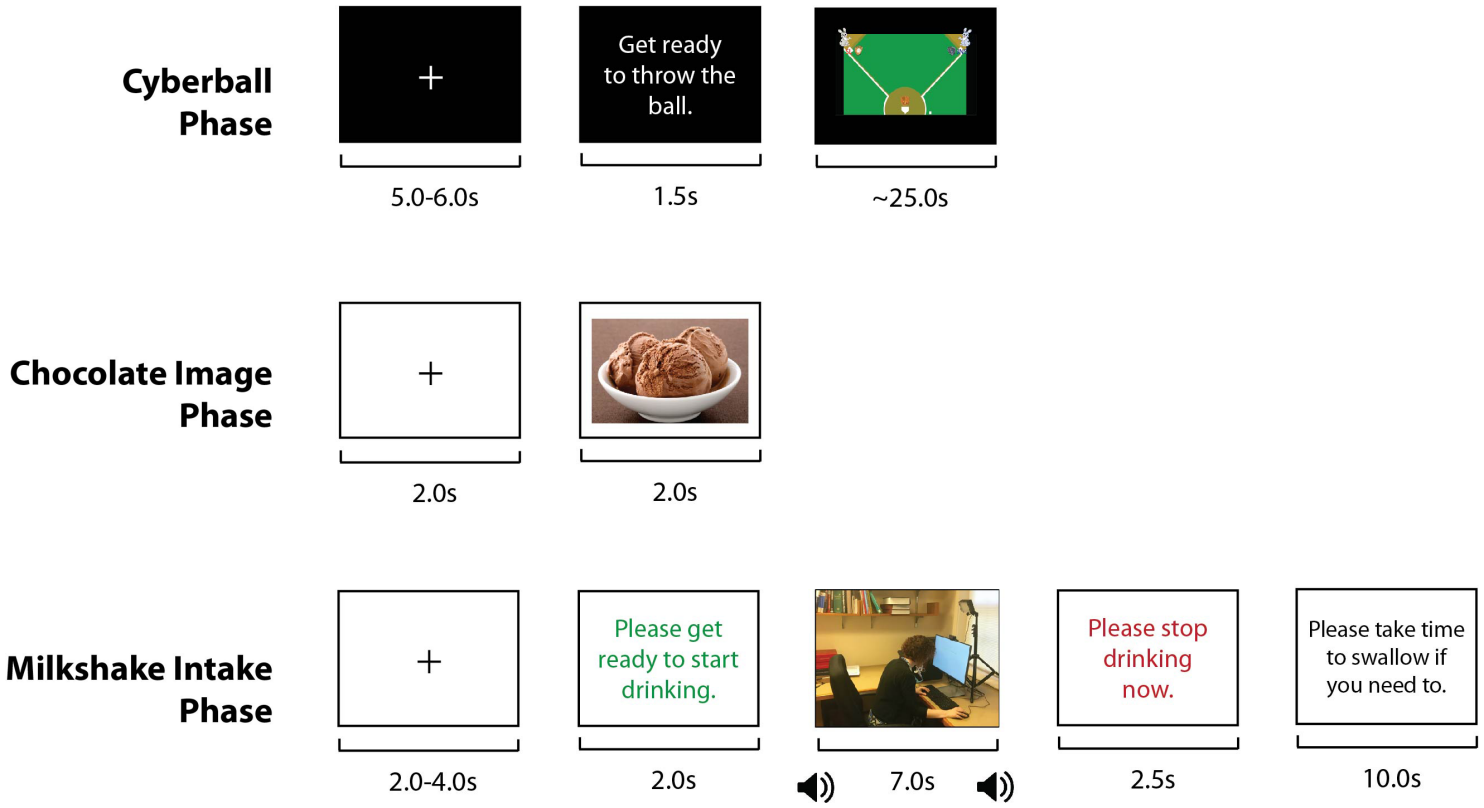
Depiction of one trial of the Cyberball-Milkshake Task. The figure illustrates the three phases of each trial. The task included 12 trials, 6 inclusion, and 6 exclusion, depending on whether the fictitious “other players” included or excluded, respectively, the participant during the Cyberball phase of the trial.

The CMT was coded in Python 3.8, using the PsychoPy package v1.84.2. Participants played 12 trials of the CMT on a desktop computer. Each trial consisted of three phases (the Cyberball, Chocolate Image, and Milkshake Intake phases; see Figure 1), each separated by viewing a fixation cross.

The 25 s Cyberball phase was based on a modified version of Cyberball designed for use with functional magnetic resonance imaging (Sebastian et al., 2011). The Cyberball phase consisted of either an exclusion round or an inclusion round of Cyberball. During exclusion rounds, the two other players never threw to the participant and threw only to each other, and during inclusion rounds, the other players over-included the participant, throwing to her with probability of 0.8 (Sebastian et al., 2011).

During the 2 s Chocolate Image phase, the participant passively viewed an image of a chocolate dessert (e.g., chocolate ice cream). Image order was randomized across participants. Since the participant could neither see nor smell the actual milkshake due to the experimental setup, the Chocolate Image Phase was included to provide a visual cue for milkshake consumption. The intent of including this phase was to better mimic real-world milkshake consumption, where milkshake cues (e.g., visual and olfactory) would be present and could potentiate ostracism’s effect on milkshake intake. Additionally, in a neuroimaging context, inclusion of the Chocolate Image phase would allow comparison of how exclusion versus inclusion affected activation in response to highly palatable food, especially given that it would likely be infeasible to analyze activation during the subsequent Milkshake Intake phase due to the confound of head motion.

During the 7 s Milkshake Intake phase, participants used a straw to consume a participant-determined amount of chocolate milkshake, the type of highly palatable food preferentially craved in response to stress and negative emotion (Domoff et al., 2014), but also avoided during dietary restriction. Milkshake intake during each trial was calculated based on the change in the milkshake’s weight, measured by a concealed digital scale.

Participants first completed a practice trial of the CMT with study staff present. During the practice trial, participants did not actually consume any milkshake, to reduce the risk of satiation during the task. Participants then completed the actual CMT while alone in the study room. The CMT comprised 6 inclusion trials and 6 exclusion trials in a pseudorandomized order, such that the same trial type (i.e., inclusion or exclusion) did not occur more than two times in a row (Sebastian et al., 2011). After completing the CMT, participants completed a final inclusion round and a final exclusion round (order counterbalanced) of Cyberball. Immediately after each final Cyberball round, the computer instructed participants to complete a packet of “task-related” questionnaires.

More detailed information about the CMT is presented in Supplementary Material Section 2.

### Interviews and Questionnaires

The interviews included the MINI International Neuropsychiatric Interview, English version 7.0.0, for DSM-5 (Sheehan, 2015), which was modified to assess both current and lifetime diagnoses of eating disorders.

The “task-related” questionnaires included: several scales assessing current affect or mood, including a mood scale containing bipolar items (e.g., happy/sad), the Implicit Positive and Negative Affect Test (IPANAT; Quirin et al., 2009), and several subscales from the Profile of Mood States 2-Adult Short Form (POMS-2-ASF; Lin et al., 2014); several manipulation check items (e.g., items assessing the intensity of ostracism experienced by the participant during the most recent round of Cyberball); and items assessing intrapersonal needs (e.g., sense of belonging) posited to be threatened by ostracism (Williams, 1997). The mood scale with bipolar items, the manipulation check items, and the need threat items were based on questions used to assess Cyberball’s impact by its developers (Williams et al., 2000; Zadro et al., 2004). Table 2 contains additional information about the task-related questionnaires, including α values.

**Table 2:** Comparison of manipulation check, affect, and need threat variables between exclusion and inclusion rounds of Cyberball.^a^

Variable name (order in which completed)	Post-inclusion round			Post-exclusion round			Post-exclusion round vs. Post-inclusion round		
	*α*	Mean	SD	*α*	Mean	SD	Paired *t*-test *t*-statistic (*p*)		*g_rm_*
Manipulation checks									
Percent Throws^b^ (8)	–	50.9	17.2	–	20.3	24.8	−5.01	(<0.001)	−1.39
Intensity of Ostracism^c^ (9)	0.94	3.3	1.7	0.89	6.4	2.1	6.11	(<0.001)	1.56
Affect									
Visual Analogue Mood^d^ (1)	0.80	6.5	1.6	0.76	6.2	1.3	−0.98	(0.34)	−0.20
IPANAT Negative Affect^e^ (6)	0.80	1.6	0.3	0.65	1.7	0.3	1.09	(0.29)	0.12
IPANAT Positive Affect^e^ (6)	0.75	2.1	0.4	0.90	2.0	0.4	−2.73	(0.02)	−0.32
POMS-2-ASF Anger-Hostility^f^ (7)	0.60	0.5	0.9	0.69	1.4	2.0	2.29	(0.04)	0.48
POMS-2-ASF Depression-Dejection^f^ (7)	0.66	0.7	1.6	0.84	0.8	1.6	0.49	(0.63)	0.06
POMS-2-ASF Tension-Anxiety^f^ (7)	0.84	1.7	2.5	0.75	1.9	2.3	0.36	(0.73)	0.06
POMS-2-ASF Vigor-Activity^f^ (7)	0.80	6.8	3.3	0.86	6.0	4.0	−1.25	(0.23)	−0.20
Need Threat									
Need Threat Belongingness^g^ (2)	–	3.0	1.5	–	6.7	1.8	6.79	(<0.001)	2.14
Need Threat Meaningful Existence^g^ (3)	–	1.5	0.7	–	1.7	1.0	1.45	(0.17)	0.19
Need Threat Control^g^ (4)	–	2.5	1.8	–	2.8	1.9	1.56	(0.14)	0.13
Need Threat Socially Oriented Self-Esteem^g^ (5)	–	4.2	1.9	–	6.0	1.7	3.75	(<0.01)	1.01

IPANAT, Implicit Positive and Negative Affect Test; POMS-2-ASF, Profile of Mood States 2-Adult Short Form; SD, Standard deviation.

a After 12 rounds of the Cyberball-Milkshake Task, participants played a final exclusion round and a final exclusion round of Cyberball (order counterbalanced); immediately after each Cyberball round ended, participants completed questionnaires about their current mood and their recent experience of Cyberball.

b Following Zadro et al. (2004), Percent Throws was the response to one item (“What percent of the throws were thrown to you?”), which was allowed to range between 0 and 100. One participant was excluded from the statistics for percent throws due to item missingness.

c Following Williams et al. (2000), Intensity of Ostracism was calculated as the mean response to items that assessed the experience of ostracism (here, “Did you feel included by the other participants?” (reverse scored); “Did you feel ignored by the other participants?”; “Did you feel excluded by the other participants?”) using a response scale ranging from 1 (Not at all) to 9 (Very much so). A higher mean response corresponds to experiencing more intense ostracism.

d Following Williams et al. (2000), the Visual Analogue Mood scale was calculated as the mean response to bipolar mood items (bad–good; happy–sad (reverse scored); tense–relaxed; rejected–accepted) using a response scale ranging from 1 (e.g., bad) to 9 (e.g., good). A higher mean response corresponds to a more positive mood.

e The IPANAT involves rating the degree to which six artificial words (e.g., VIKES) express three negative moods (helpless, tense, and inhibited) and three positive moods (happy, cheerful, and energetic), using response options ranging from “Does not fit at all” (=1) to “Fits very well” (=4). Scores for Negative Affect and Positive Affect are calculated by first averaging responses regarding each mood across the six artificial words, and then averaging the resulting mood scores across the three negative and three positive moods, respectively. One participant was excluded from the statistics for both Negative Affect and Positive Affect due to item missingness.

f Items from four POMS-2-ASF scales (Anger-Hostility; Depression-Dejection; Tension-Anxiety; Vigor-Activity) were administered. For each item, participants indicated the degree to which they were experiencing a particular feeling using response options including “Not at all” (=0), “A little” (=1), “Moderately” (=2), “Quite a bit” (=3), and “Extremely” (=4). Scores for each scale were calculated by summing responses to items belonging to that scale, with a higher score corresponding to greater endorsement of the relevant mood state.

g Similar to Williams et al. (2000), Need Threat was the response to items that assessed threat to certain intrapersonal needs posited to be threatened by ostracism (Belongingness: “How much do you feel you belonged to the group?” (reverse scored); Meaningful Existence: “How true is the statement: ‘Life is meaningless’?”; Control: “How true is the statement: ‘I am in control of my life’?” (reverse scored); Socially Oriented Self-Esteem: “To what extent do you think the other participants valued you as a person?” (reverse scored)) using a response scale ranging from 1 (“Not at all”) to 9 (“Very much so”). Responses to certain items were reversed so that higher responses correspond to greater need threat.

The “final” questionnaires included the DEBQ, which includes an Emotional Eating scale (12 items; e.g., “Do you have a desire to eat when you are depressed or discouraged?”). Responses are indicated on a 5-point scale ranging from “Never” (=1) to “Very Often” (=5). In our sample, *α* = 0.97 for the DEBQ Emotional Eating scale.

The funnel debriefing interview (see Supplementary Material Section 3) was adapted from Lakin (2003). Supplementary Material Section 4 presents information on interview response coding and awareness of critical aspects of the study.

### Data Analysis

All analyses were conducted in R version 4.0.2. Planned analyses were specified *a priori*, and exploratory analysis were specified based on results of planned analyses.

#### Planned Analyses

To examine the effects of Cyberball condition (exclusion vs. inclusion) on self-report measures, we performed paired *t*-tests and calculated Hedges’ *g_rm_* as a measure of effect size (Lakens, 2013). We also performed Wilcoxon signed rank tests for paired samples, but did not report results because they were very similar to those of paired *t*-tests. To examine the effects of Cyberball condition on milkshake intake, we fitted two linear mixed effects models to the trial-level data using the nlme package for R: Model 1 examined the effect of exclusion (vs. inclusion) in the sample overall, and Model 2 examined how the effect of exclusion (vs. inclusion) differed based on DEBQ Emotional Eating scores. In all models, the outcome variable was milkshake intake (in g), centered around the participant-specific mean. Predictors in Model 1 were trial-level variables that might influence milkshake intake, including: indicators for the chocolate images that preceded milkshake intake; Trial and Trial^2^, with Trial equal to the trial number minus 6.5; and an indicator for Condition (exclusion = 1; inclusion = 0). (We used AIC to compare linear, quadratic, and cubic parameterizations for Trial, which indicated that the quadratic model fit best.) Predictors in Model 2 included all Model 1 predictors, as well as a main effect for DEBQ Emotional Eating and its interaction with the indicator for Condition. Both models included a random intercept at the participant level.

#### Exploratory Analyses

Supplementary Material Section 5 presents information on exploratory analyses, as well as a description of questionnaires included in those analyses.

## Results

This section presents results from planned analyses, and Supplementary Material Section 5 presents results of exploratory analyses.

### Sample

For the 20 participants included in the analysis sample, the mean (SD) age was 23.1 (3.8) years. Table 1 presents additional demographic and other information for these participants.

The 11 participants reporting high emotional eating at screening showed a substantial prevalence of lifetime major depressive disorder (54.5%) and eating disorders, with 27.3% meeting criteria for a current eating disorder and 45.5% meeting criteria for a past eating disorder. (One individual met criteria for both bulimia nervosa and binge-eating disorder at different points in the past.) In contrast, of the 9 participants reporting low emotional eating at screening, zero and 11.1% met criteria for a current or past eating disorder, respectively.

### Effects of Cyberball Exclusion on Self-Report

In “task-related” questionnaires administered after the final exclusion and inclusion rounds of Cyberball, participants reported receiving a significantly smaller percentage of throws and feeling significantly more intense ostracism after exclusion, compared to inclusion (Table 2). Also, participants reported significantly greater threat to belongingness and to socially oriented self-esteem after exclusion, compared to inclusion; however, threat to meaningful existence and to control did not differ between exclusion and inclusion rounds. Effect sizes for percent throws, intensity of ostracism, threat to belongingness, and threat to self-esteem were large. Finally, although changes in affect were all in the expected direction (i.e., greater negative, and less positive, affect after the exclusion round), they were generally small and non-significant.

### Milkshake Intake

**Figure 2:**
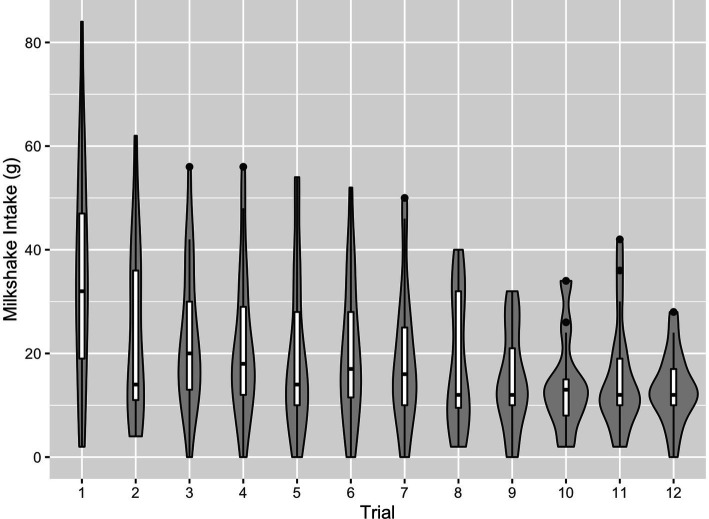
Violin plots of milkshake intake, by trial. Violin plots reveal that median milkshake intake was highest during the first trial and generally decreased thereafter, with reduced variability in later trials.

On 4.2% of trials, milkshake intake could not be calculated due to missing values, or calculations produced impossible values of negative intake. Figure 2 depicts the distribution of calculated milkshake intake values for each trial. Intake was markedly higher for the first trial and declined thereafter, and there was reduced variability for later trials.

### Effect of Cyberball Exclusion on Milkshake Intake

**Figure 3:**
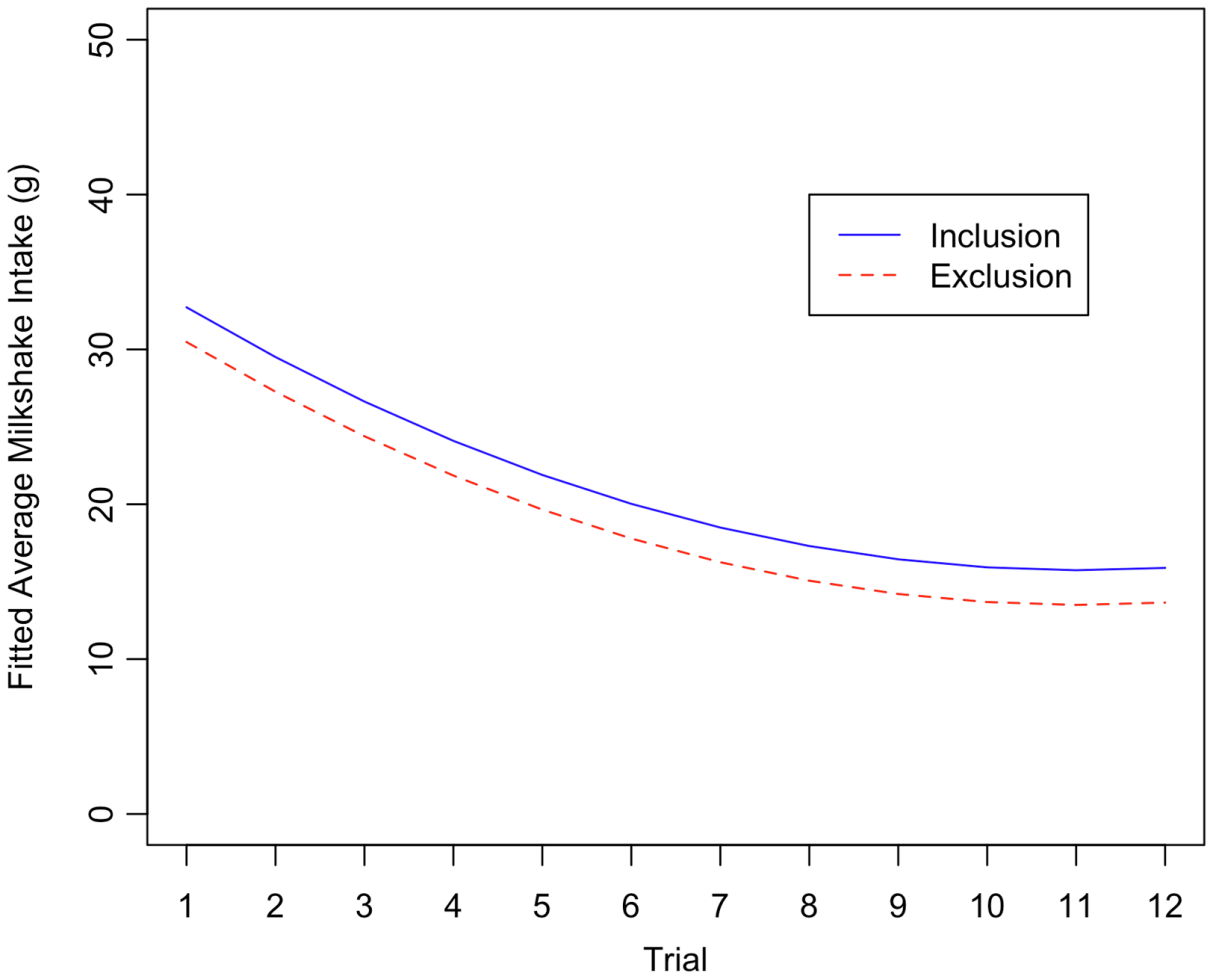
Plot of fitted values of milkshake consumption versus trial number, by condition. Fitted values were calculated from the results of Model 1, for an average participant’s chocolate milkshake intake and for an average chocolate image.

Table 3 present results for linear mixed effects models. In Model 1, the main effect of Condition was −2.24 (95% CI [−4.76, 0.28]), which is in the direction of reduced milkshake intake after exclusion (vs. inclusion; see Figure 3), although the confidence interval includes small positive values. In Model 2, the interaction between Condition and DEBQ Emotional Eating was −1.11 (95% CI [−3.36, 1.13]), which is in the opposite direction as expected, although the confidence interval does include positive values. In both models, milkshake intake decreased quadratically across trials, with a steep negative slope for low trial numbers that decreased to the point of being flat for the highest trial numbers.

**Table 3:** Model fitting results for Cyberball-Milkshake Task (all trials).^a^

	Model 1^b^		Model 2^c^	
	Estimate	95% CI	Estimate	95% CI
Intercept	−3.17	[−7.75, 1.41]	−4.77	[−10.94, 1.40]
Chocolate Image: FP0289	−0.21	[−6.23, 5.81]	−0.16	[−6.20, 5.88]
Chocolate Image: FP0675	1.52	[−3.57, 6.61]	1.60	[−3.50, 6.71]
Chocolate Image: FP0703	3.12	[−2.01, 8.24]	3.14	[−2.00, 8.28]
Chocolate Image: FP0713	2.04	[−3.91, 7.99]	2.27	[−3.71, 8.25]
Chocolate Image: FP0083	0.78	[−5.16, 6.73]	0.94	[−5.03, 6.91]
Chocolate Image: FP0878	1.21	[−4.75, 7.17]	1.42	[−4.57, 7.41]
Chocolate Image: FP0879	4.68	[−1.29, 10.65]	5.19	[−0.88, 11.26]
Chocolate Image: IAPS7330	4.97	[−1.04, 10.98]	5.23	[−0.82, 11.27]
Chocolate Image: IAPS7340	5.12	[−0.80, 11.04]	5.39	[−0.57, 11.35]
Trial	−1.53	[−1.89, −1.17]	−1.52	[−1.89, −1.16]
Trial^2^	0.17	[0.05, 0.29]	0.16	[0.04, 0.28]
Condition: Exclusion	−2.24	[−4.76, 0.28]	0.63	[−5.68, 6.94]
DEBQ EE	–	–	0.59	[−1.07, 2.26]
DEBQ EE × Condition: Exclusion	–	–	−1.11	[−3.36, 1.13]

CI, Confidence interval; DEBQ EE, Dutch Eating Behavior Questionnaire Emotional Eating; FP, FoodPics; IAPS, International Affective Picture System; SE, Standard error

a Outcome variable is milkshake intake in grams, centered based on subject-specific mean milkshake intake.

b Model 1 predictors include Chocolate Image (treated as a dummy coded categorical variable, with FP0167 as the reference category), Trial and Trial^2^ (with Trial treated as a continuous variables and centered around 6.5), and Condition (treated as dummy coded categorical variable, with Inclusion as the reference category).

c Model 2 predictors include all Model 1 predictors, DEBQ EE (treated as a continuous variable), and DEBQ EE × Condition.

## Discussion

We introduced the cyberball-milkshake task (CMT), which is designed to measure ostracism’s impact on the consumption of highly palatable food. Our goal was to develop a task that would facilitate future investigations of individual differences in ostracism’s effects on eating behavior, including potential neuroimaging investigations into the neural underpinnings of these effects (Sebastian et al., 2011). The resulting task could be used to investigate ostracism’s effect on eating behavior in the general population. Additionally, given evidence that negative interpersonal experiences may precipitate eating disorder symptoms, such as binge-eating and restriction (Rieger et al., 2010), and that ostracism and rejection are especially negative interpersonal experiences (Baumeister et al., 2005; Williams, 2007), particularly for individuals with eating disorders (Cardi et al., 2013), the task might also prove useful for investigating how negative interpersonal experiences precipitate certain eating disorder symptoms (e.g., binge-eating and restriction) in samples with eating disorders. Additionally, the task could also be used in samples with eating disorders to examine whether current eating disorder interventions are efficacious at mitigating ostracism’s effects on eating behavior. If feasible and valid, the task could fill an important gap since few studies have examined the impact of interpersonal stressors on actual eating behavior in eating disorders (Monteleone et al., 2018), despite the importance of rigorous and reproducible laboratory studies of eating behavior for advancing our understanding of eating behavior and eating disorders (Sysko et al., 2018).

In the present study, we examined the feasibility of the CMT in a sample of individuals who reported very high or very low levels of emotional eating at screening. We also preliminarily examined the effects of ostracism on milkshake intake in the overall sample, and among individuals with varying levels of self-reported emotional eating. The following discussion focuses on results of planned analyses, except for a few instances (noted below) where we refer to results of exploratory analyses.

Regarding feasibility, exclusion during Cyberball generally had the intended effect, except for a potentially smaller-than-expected effect on affect. As expected, participants perceived receiving fewer throws during exclusion than inclusion, giving estimates comparable to prior studies using the modified version of Cyberball (Sebastian et al., 2011). Further, even after playing twelve prior rounds of Cyberball, exclusion during Cyberball led to greater feelings of ostracism and threatened psychological needs of belonging and socially oriented self-esteem, when compared to inclusion. Notably, prior research examining Cyberball’s impact on the same need threat items also found that greater ostracism threatened belonging and self-esteem, but not control and meaningful existence (Williams et al., 2000). Finally, exclusion during Cyberball generally had small and non-significant effects on affect, whether assessed *via* implicit or explicit self-report measures. Importantly, some prior research (including a study with Cyberball) suggests that ostracism and rejection have limited effects on mood and that their effect on behavior, including eating behavior, is not mediated *via* changes in mood (Baumeister et al., 2005; Oaten et al., 2008). In contrast, a number of other studies have demonstrated higher levels of sadness and anger during Cyberball exclusion, at least as reported retrospectively (Williams, 2007). It is possible that exclusion would have had a greater impact on affect measured during Cyberball (rather than afterward), especially during an earlier trial of the task. It is also possible that Cyberball exclusion impacts affect only in certain subgroups (e.g., individuals high on rejection sensitivity), which our study was not powered to detect. Finally, our results and prior research (Baumeister et al., 2005) could also suggest another possibility, which is that negative interpersonal experiences are not necessarily accompanied by notable changes in subjective affect. If that is the case, increases in subjective negative affect may not fully mediate the link between negative interpersonal experiences and eating behavior, including eating disorder symptoms (Rieger et al., 2010).

Also regarding feasibility, milkshake intake was measurable for more than 95% of participants, and on more than 95% of the trials for those participants. Intake varied considerably across participants, especially in the earlier trials. Further, intake decreased sharply across initial trials and remained low and relatively constant across the final trials. This pattern raises the possibility that general or sensory-specific satiety affected intake during later trials. Future studies with the CMT should consider using fewer trials, and potentially excluding intake during the first trial from analysis, since it is markedly higher and potentially more influenced by factors not of interest, such as hunger.

Awareness checks (see Supplementary Material Section 4) revealed that between 40 and 65% of participants reported being aware during the CMT that the “other players” were not real, despite efforts to make participants believe they were playing against other study participants (see Supplementary Material Section 2). This number is higher than reported for other studies using Cyberball (Kelly et al., 2012). It is possible that our study design, specifically the repetition of shorter inclusion and exclusion rounds, may have increased participants’ awareness that Cyberball was computerized or scripted (Best, 2010). However, this finding should be considered in light of research suggesting that Cyberball threatens psychological needs even when participants are aware they are playing against the computer or that the other players’ behavior is scripted (Zadro et al., 2004), as well as research suggesting that Cyberball impacts physiological measures (e.g., skin conductance) even among “suspicious” participants (Kelly et al., 2012). In contrast, a minority of participants reported being aware that their milkshake intake was being measured, or that the CMT’s aim was to assess the effect of ostracism on milkshake intake, during the CMT. These percentages compare favorably to similar laboratory studies (e.g., of emotional eating), where the majority of participants were aware that experimenters were assessing their food intake (Altheimer et al., 2021).

In our first model for milkshake intake, Cyberball exclusion was estimated to have a small negative effect on intake, although it is important to note that the 95% confidence interval for this effect included very small positive values. Prior research has sometimes, but not always, found greater intake of sweet food after Cyberball exclusion (Oaten et al., 2008; Hayman et al., 2015). However, there are certain methodological differences between this prior laboratory research and the CMT that could alter exclusion’s effects on milkshake intake. For one, the CMT used shorter rounds of Cyberball, which may have had less of a potentiating effect on intake of highly palatable food. Additionally, in the CMT, the Milkshake Intake phase occurred seconds after the end of the Cyberball phase, rather than following an intervening questionnaire (Oaten et al., 2008); it may be that a longer delay between exclusion and milkshake intake, longer access to the milkshake, or being asked questions about the exclusion experience would allow greater reflection on the ostracism experience and result in greater consumption as a coping response (Hayman et al., 2015). Also, and not mutually exclusively, it is possible that, for some individuals, the immediate response to ostracism is reduced desire for highly palatable foods or an impulse to restrict one’s intake of highly palatable foods.

In our second model for milkshake intake, Cyberball exclusion did not have the expected positive interaction with self-reported emotional eating, as measured by DEBQ Emotional Eating scores, although it is important to note that the 95% confidence interval for this interaction did include positive values. In evaluating these results, it is important to consider that our sample size was small, that there was some restriction of range due to the absence of participants with extremely high Emotional Eating scores, and that Cyberball exclusion had a relatively small impact on negative affect. However, even in the absence of these study features, current evidence suggests that self-reported emotional eating would be unlikely to strongly predict task performance. At the time the present study was planned (2015–2016), research suggested that very high and very low Emotional Eating scores predicted eating behavior (van Strien, 2010), although some researchers had already begun to question the validity of self-reported emotional eating (Evers et al., 2009; Domoff et al., 2014). Since then, additional evidence has accumulated to suggest that self-report measures of emotional eating may have limited validity (Bongers and Jansen, 2016; Evers et al., 2018; Altheimer et al., 2021).

Our study has several strengths, including a successful method of inducing ostracism and objective measurement of food intake. Further, our study adheres to many methodological best practices for laboratory studies of eating behavior (Robinson et al., 2018), including efforts to standardize pre-task food consumption, efforts at blinding participants to the measurement of food intake and study aims, and measurement of blinding success.

The study also has certain limitations that should be considered. First, the sample size was small, which limits confidence in results. Also, although we used a measure of effect size more appropriate to small samples, effect size may still have been overestimated (Lakens, 2013). Additionally, we did not correct for multiple comparisons (e.g., when examining the effect of exclusion on affect), although we took care not to interpret isolated significant or trend level results. However, the CMT did have six trials per condition for each participant, yielding higher power than a single instance of each condition per participant, or a between-participant design; additionally, our sample size was above the median for within-person studies of eating behavior (Robinson et al., 2018). Another limitation is that the sample was comprised of young adult women, and the majority of participants identified as White; these demographic groups are over-represented in eating disorder research, and future research should employ more diverse samples (Goel et al., 2022). Additionally, the CMT Cyberball phase was based on a modified version of Cyberball that contrasts exclusion with over-inclusion (as opposed to equal inclusion), to ensure that participants perceive the difference between conditions given the shorter rounds used in the modified version (Sebastian et al., 2011). Use of over-inclusion can complicate interpretation of differences (e.g., in milkshake intake) between conditions, raising the question of whether differences are due to the negative experience of exclusion or to the more positive experience of over-inclusion (De Waal-Andrews and Van Beest, 2020). Notably, in the present study, exploratory analyses found that individuals who reported being more sensitive to rejection also experienced greater threats to socially oriented self-esteem for exclusion minus inclusion (see Supplementary Material Section 5), suggesting that lower self-esteem after exclusion may be due at least in part to a detrimental effect of exclusion on self-esteem. However, this does not preclude effects of over-inclusion on psychological variables, such as self-esteem, or behavioral variables, such as milkshake intake. For example, over-inclusion could impact milkshake intake by inducing positive emotions, which have been shown to affect food intake (Cardi et al., 2015; Evers et al., 2018). Finally, the inclusion of a Chocolate Image phase between the Cyberball and Milkshake Intake phases of the CMT may have introduced extraneous variability into participants’ milkshake intake, depending on how external food cues impacted their behavior.

These limitations notwithstanding, the present study suggests that the CMT is generally a feasible approach to examining how ostracism affects consumption of highly palatable food. The present study also suggests several ways in which future users of the CMT could consider modifying task design and analysis to potentially improve feasibility and validity. First, reducing the number of CMT trials may make it less likely that participants will habituate to the effects of Cyberball exclusion or begin to suspect that the game is computerized or scripted. Further, doing so may reduce the likelihood of general or sensory-specific satiety impacting milkshake intake. Additionally, given that milkshake intake on the first CMT trial appears to be markedly higher and is potentially more influenced by factors not of interest (e.g., hunger), it may be advisable to exclude the first trial from analysis. Second, users may wish to consider longer rounds of Cyberball, as in the more standard version of the game (Williams et al., 2000), since longer rounds may potentiate the experience of exclusion and lead to more marked effects of exclusion on milkshake intake. Longer rounds would also facilitate contrasting exclusion with (equal) inclusion, as opposed to over-inclusion, a third potential modification that would simplify interpretation of differences (e.g., in milkshake intake) for exclusion vs. inclusion. Fourth, omitting the Chocolate Image phase may reduce extraneous variability (e.g., due to the effects of food cues) on milkshake intake. Fifth, with regards to analysis of milkshake intake, Condition (i.e., exclusion vs. inclusion) could be allowed to interact with the high-order terms for trial number, rather than assuming that Condition has a constant effect on milkshake intake regardless of trial number.

Additionally, careful consideration should be given to how best to validate the CMT in future research. Although we used a questionnaire measure of emotional eating in response to general negative affect (specifically, the DEBQ Emotional Eating scale) to do so, this may not have been the best approach to validation, for multiple reasons. For one, ostracism may not induce marked changes in subjectively experienced emotions (Baumeister et al., 2005; Oaten et al., 2008), suggesting that measures of how individuals’ food intake changes in response to ostracism, rather than in response to negative emotions, may better predict CMT performance. Additionally, questionnaire measures may not accurately capture how various factors (e.g., negative experiences) actually influence individual’s eating behavior in the natural environment (Bongers and Jansen, 2016), suggesting it may be better to focus on comparing CMT performance to ecological momentary assessment of naturalistic behaviors, which may be subject to fewer biases (e.g., in recall) than questionnaires (Reichenberger et al., 2020). Further, more specific, as opposed to more general, measures may be more likely to predict behavior on the CMT (Sproesser et al., 2014), suggesting that CMT performance may be better predicted by measures focusing on changes in individuals’ food intake in response to ostracism in particular, rather than in response to negative experiences more generally. Finally, measures that assess decreases as well as increases in actual food intake may be more likely to predict eating behavior on the CMT, compared to measures, such as the DEBQ Emotional Eating scale, that focus on assessing the *desire* to eat and that focus only on increases (Meule et al., 2018; Reichenberger et al., 2020). Given these reasons, our general recommendation would be to validate the CMT with respect to ecological momentary assessment focused on measuring the association between interpersonal stressors (or potentially ostracism in particular) and intake of highly palatable food in the natural environment (O’Connor et al., 2008). Additionally, if the CMT were to be validated with respect to a questionnaire measure, our recommendation would be to use a measure of restrained eating (Reichenberger et al., 2020), which has been shown to predict behavior in response to negative experiences in experimental studies (Evers et al., 2018). If the CMT were to be validated with respect to a questionnaire-based measure of emotional eating, our recommendation would be to use the Salzburg Emotional Eating Scale (Meule et al., 2018), which assesses changes in food intake, including reduced intake, in response to specific emotions. Notably, in our exploratory analyses of CMT Trials 2 through 7 (see Supplementary Material Section 5), individuals who reported eating less in response to anxiety (on the Anxiety subscale of a modified Emotional Eating Scale) demonstrated reduced milkshake intake following exclusion, whereas individuals who reported eating more in response to anxiety demonstrated increased milkshake intake following exclusion.

Finally, regarding sample considerations, future research should investigate the CMT in a considerably larger sample that includes individuals with and without eating disorders (Reichenberger et al., 2020).

## Data Availability Statement

The raw data supporting the conclusions of this article will be made available by the authors, without undue reservation.

## Ethics Statement

The studies involving human participants were reviewed and approved by MassGeneral Brigham Institutional Review Board. The patients/participants provided their written informed consent to participate in this study.

## Author Contributions

KNJ conceived, designed the study, and performed the statistical analysis and wrote the first draft of the manuscript. HGP, JIH, SAG, and SFG contributed to the design of the study. KNJ, EML, LLP, MER, and CP acquired the data for the study. All authors contributed to manuscript revision and approved the submitted version.

## Funding

The present study was funded by the Phyllis and Jerome Lyle Rappaport Foundation Mental Health Research Scholar Award at McLean Hospital. KNJ was also supported by the National Institute of Diabetes and Digestive and Kidney Diseases of the National Institutes of Health (Award Number K23-DK120517) and by the Martini Family Foundation, Nancy Dearman, and the McLean Hospital Women’s Mental Health Innovation Fund. The content is solely the responsibility of the authors and does not necessarily represent the official views of the National Institutes of Health.

## Conflict of Interest

JIH has received consulting fees from Idorsia, Otsuka, and Sunovion and has received research grant support from Boehringer-Ingelheim, Idorsia, and Sunovion.

The remaining authors declare that the research was conducted in the absence of any commercial or financial relationships that could be construed as a potential conflict of interest.

## Publisher’s Note

All claims expressed in this article are solely those of the authors and do not necessarily represent those of their affiliated organizations, or those of the publisher, the editors and the reviewers. Any product that may be evaluated in this article, or claim that may be made by its manufacturer, is not guaranteed or endorsed by the publisher.

## Footnotes

^1^ https://rally.partners.org
