# Supplementary material for: Measuring Ostracism-Induced Changes in Consumption of Palatable Food: Feasibility of a Novel Behavioral Task
Source: Front Psychol. 2022 May 17;13:853555. doi: 10.3389/fpsyg.2022.853555 (PMC9157248; doi:10.3389/fpsyg.2022.853555)
Supplement: Supplementary file 1 [file Data_Sheet_1.docx]

Supplementary Material

# Additional Eligibility Criteria

Additional inclusion criteria included: being born and raised primarily inside the United States, since responses to ostracism can vary between cultures (Uskul and Over, 2017); and being able and willing to consume chocolate milkshake and rating one’s overall impression and liking/enjoyment of the milkshake as ≥5 on a visual analogue scale ranging from 1 = “Extremely bad” to 7 = “Extremely good.”

Exclusion criteria included: recent suicidality or self-harm; substantial experience studying or working in psychology or related fields, or working in social science or biomedical research; presence of major lifetime medical condition (e.g., Type 1 or 2 diabetes) or current illness (e.g., upper respiratory infection) that would affect performance on CMT; lifetime history of mild or major DSM-5 neurocognitive disorders, neurological problem (e.g., stroke), or moderate to severe head injury; lifetime DSM-5 intellectual disability, autism spectrum disorder, or communication, learning, or motor disorder of severity sufficient to interfere with completion of study protocol; lifetime DSM-5 schizophrenia spectrum or other psychotic disorders, bipolar or related disorders, or depressive disorder with psychotic features; other DSM-5 psychiatric (including substance use disorder) symptoms of severity sufficient to interfere with completion of study protocol; recent (i.e., past twelve month) DSM-5 anorexia nervosa or body mass index ≤17; recent use of medications that are psychotropic or affect eating (e.g., prednisone); and recent initiation of psychotherapy or counseling focused on addressing emotions or eating.

# Detailed Information on Cyberball-Milkshake Task

As is standard for Cyberball, participants were informed that our interest lay in mental visualization, to conceal the fact that Cyberball seeks to manipulate social interactions. Specifically, the CMT instructions (see Table S2.1) stated that the CMT’s aim was to test the effects of mental visualization on taste. (The task-related questionnaires, which were completed at the end of the task, contained questions about the milkshake’s taste, among other questions.) Also, we used several strategies to increase the likelihood that participants would believe the “other players” in Cyberball were real. After a staff member finished instructing the participant, the staff member called another staff member to inquire if they were “ready on their end” to begin the practice trial. Additionally, at the start of the practice trial, the participant was asked to select a number from 1 to 100 and then informed that, based on their selection, they would be Player 1, who would start each round with the ball. Subsequently, each player was introduced, in order, by their initials, and these initials appeared next to their avatar during Cyberball rounds. Finally, the task was programmed so that the “other players” took a variable amount of time (0.8-3.1s), and occasionally much longer (3.8 – 6.1s), before throwing the ball, as if deciding to whom to throw next (Sebastian et al., 2011).

The 25s Cyberball phase of the task was based on a modified version of Cyberball (Sebastian et al., 2011) that consists of several shorter inclusion and exclusion rounds in a pseudorandomized order. In comparison to using a longer inclusion round followed by a longer exclusion round, the modified version reduces concerns about expectation violation as a confound of ostracism (Somerville et al., 2006). Also, based on feedback regarding lack of engagement from participants during initial piloting with the original black-and-white line drawing interface for Cyberball, we used an alternative, more colorful interface (Nieuwburg, 2015). This interface depicts a baseball diamond with gloves representing Players 1, 2, and 3, and it includes sounds when the ball is thrown. Rather than representing the other players with photographs, which could introduce additional variance depending on participants’ past interpersonal experiences, other players were represented by animal cartoon avatars. During exclusion rounds, the two other players never threw to the participant, and during inclusion rounds, the other players over-included the participant, throwing to her with probability of 0.8 (Sebastian et al., 2011). Also, the participant was instructed to click on the mitt of the other player to whom she wished to throw (if the participant had the ball) or her own mitt (if another participant had the ball), to better equate motor actions and engagement across inclusion and exclusion rounds (Sebastian et al., 2011).

During the 2s Chocolate Image phase of the task, the participant passively viewed an image of a chocolate dessert (e.g., chocolate ice cream). Images were selected from *food-pics* (Blechert et al., 2014, 2019; Image numbers 0083, 0167, 0289, 0675, 0703, 0713, 0878, 0879) and the International Affective Picture System (Lang et al., 2008; Image numbers 7330 and 7340). Image order was randomized across participants. Since the participant could neither see nor smell the actual milkshake due to the experimental setup, the Chocolate Image Phase was included to provide a visual cue for milkshake consumption. The intent of including this phase was to better mimic real-world milkshake consumption, where milkshake cues (e.g., visual, olfactory) would be present and could potentiate ostracism’s effect on milkshake intake. Additionally, in a neuroimaging context, inclusion of the Chocolate Image phase would allow comparison of how exclusion versus inclusion affected activation in response to highly palatable food, especially given that it would likely be infeasible to analyze activation during the subsequent Milkshake Intake phase due to the confound of head motion.

During the 7s Milkshake Intake phase of the task, participants used a straw to consume chocolate milkshake, the type of palatable food preferentially craved in response to stress and negative emotion (Domoff et al., 2014), but also avoided during dietary restriction. Participants were instructed to start drinking when they heard an initial tone and to consume at least enough milkshake to experience the taste, and they were informed that they could continue drinking until they heard a second tone. The milkshake (3 servings of a packaged, commercially available chocolate milkshake) was contained in a large, insulated mug; the total amount of milkshake available for consumption was 48 fl oz, totaling 1740 kcal (Total Fat: 69g; Total Carbohydrates: 243g; Protein: 36g). The mug sat inside an opaque box, on top of a digital scale; to provide a rationale for placing the mug in a box, study staff informed participants that, if the milkshake spilled, the box would protect nearby technical equipment. The digital scale was connected (via concealed cords) to the task computer, and the task computer read the weight from the scale once before the participant began drinking (“measurement 0”) and then again every second between the initial and final tone (“measurements 1-7”). Milkshake intake during Trial *k* (for *k* = 1, . . . 11) was subsequently calculated by subtracting measurement 0 for Trial *k* + 1 from measurement 0 for Trial *k*; milkshake intake during Trial 12 (the final CMT trial) was calculated by subtracting measurement 7 for Trial 12 from measurement 0 for Trial 12.

| **Table S2.1. Cyberball-Milkshake Task Instructions** |
| --- |
| In the upcoming experiment, we test the effects of practicing mental visualization on taste. Thus, we need you to practice your mental visualization skills. We have found that the best way to do this is to have you play an online ball tossing game with other participants who are logged in at the same time.  In a few moments, you will be playing a ball tossing game with two other female participants your age over the network. The game is very simple. When the ball is tossed to you, simply left-click on the baseball mitt in front of the player you want to throw it to. Also, to make sure you're keeping track of the ball when another player has it, please left-click (once) on your own baseball mitt while the other player decides where to throw the ball.  What is important is not your ball tossing performance, but that you MENTALLY VISUALIZE the entire experience. Imagine what the others look like. What sort of people are they? Where are they playing? Is it warm and sunny or cold and rainy? Create in your mind a complete mental picture of what might be going on if you were playing the game in real life.  You will play the game for about 10 minutes, and every so often you will all be given a break where you will first view a picture of chocolate and then drink the milkshake. You'll be told when you should GET READY to start drinking. A tone will then tell you when you can actually start drinking. Please drink AT LEAST ENOUGH MILKSHAKE TO EXPERIENCE THE TASTE (i.e., at least one sip). You can keep drinking until the second tone tells you to stop. Please drink from the milkshake ONLY when told to do so.  Whenever you're not playing the ball tossing game or drinking the milkshake, a cross will appear in the middle of the screen. Please keep your focus on the cross. |

# Funnel Debriefing Interview

**Part I (administered immediately after Cyberball-Milkshake Task):**

I.3) *What do you think the point of the task was? In other words, what was the point of playing the throwing game and drinking the chocolate milkshake?*

IF THE PARTICIPANT GIVES A DEFINITIVE ANSWER:

*At what point did you start thinking that? – Was it while you were playing the game, after you completed the questionnaires at the end of the game, etc?*

I.4a) *When you were playing the throwing game, did anything stand out to you? (If so, what?)*

IF THE PARTICIPANT GIVES A DEFINITIVE ANSWER:

*At what point did you start thinking that? – Was it while you were playing the game, after you completed the questionnaires at the end of the game, etc?*

IF SUBJECT MENTIONS EXCLUSION IN I.4a:

I.4b) *Why do you think that happened?* (i.e., *Why do you think you were excluded?*)

IF THE PARTICIPANT GIVES A DEFINITIVE ANSWER:

*At what point did you start thinking that? – Was it while you were playing the game, after you completed the questionnaires at the end of the game, etc?*

I.5) *When you were playing the throwing game, who did you think you were playing the throwing game with?*

IF THE PARTICIPANT GIVES A DEFINITIVE ANSWER:

*At what point did you start thinking that? – Was it while you were playing the game, after you completed the questionnaires at the end of the game, etc?*

I.6a) *When you were playing the throwing game, what do you think we were looking at while you were drinking the milkshake, if anything?*

IF THE PARTICIPANT GIVES A DEFINITIVE ANSWER:

*At what point did you start thinking that? – Was it while you were playing the game, after you completed the questionnaires at the end of the game, etc?*

IF SUBJECT MENTIONS MEASURING AMOUNT IN I.6a:

I.6b) *Why do you think we were doing that? ?* (i.e., *Why do you think we would measure the amount you were drinking?*)

IF THE PARTICIPANT GIVES A DEFINITIVE ANSWER:

*At what point did you start thinking that? – Was it while you were playing the game, after you completed the questionnaires at the end of the game, etc?*

**Part II (administered immediately before study debriefing):**

II.1) *Are you wondering anything about this research study, or do you have any questions about it?*

II.2) *What did you think this research study was about?*

IF THE PARTICIPANT GIVES A DEFINITIVE ANSWER:

*At what point did you start thinking that? – Was it while you were playing the game, after you completed the questionnaires at the end of the game, etc?*

II.3) *What do you think the point of the task was? In other words, what was the point of playing the throwing game and drinking the chocolate milkshake?*

II.4a) *When you were playing the throwing game, did anything stand out to you? (If so, what?)*

IF SUBJECT MENTIONS EXCLUSION in II.4a:

II.4b) *Why do you think that happened?* (i.e., *Why do you think you were excluded?*)

IF SUBJECT DOES NOT MENTION EXCLUSION in II.4a:

II.4c) *Did you receive the ball more often or less often than the other players?*

IF YOU ASKED II.4c, ALSO ASK:

II.4d) *Why do you think that happened?* (i.e., *Why do you think you were excluded?*)

II.5) *When you were playing the throwing game, did you think the other participants were real people?*

II.6a) *What do you think we were measuring when you were drinking the milkshake?*

IF SUBJECT MENTIONS MEASURING AMOUNT in II.6a:

II.6b) *Why do you think we were doing that?* (i.e., *Why do you think we would measure the amount you were drinking?*)

IF SUBJECT DOES NOT MENTION MEASURING AMOUNT in II.6a:

II.6c) *Did you think we were measuring how much milkshake you were drinking?*

IF YOU ASKED II.6c, ALSO ASK:

II.6d) Why do you think we were doing that? (i.e., *Why do you think we would measure the amount you were drinking?*)

II.7) *What do you think we were trying to find out about you from all the surveys you filled out?*

# Funnel Debriefing Interview and Awareness Checks

The funnel debriefing interview (see Appendix C) was administered in two phases (see Procedures), and responses were transcribed verbatim. Two or more study staff used the transcribed responses to code whether each participant was aware of certain critical aspects of the study (see Table S4.1, column 1) and, if so, when they become aware (i.e., during the CMT, after the CMT, or at a time that could not be determined). Discrepancies in coding were resolved through discussion with the Principal Investigator. When tallying results for awareness, participants who became aware at an indeterminate time were included with those who reported becoming aware during the CMT, to provide conservative estimates of awareness during the CMT.

Table S4.1 presents statistics pertaining to awareness of critical study aspects. Between 40% and 65% of participants reported awareness at some point during the CMT that the other Cyberball participants were not real. However, only 5% to 25% reported awareness during the CMT that their milkshake intake was being measured, and only 5% to 15% reported awareness during the CMT that the task’s aim was to assess how ostracism (or negative emotion) impacted milkshake intake. An additional 10%, 30%, and 40%, respectively, reported becoming aware of these aspects of the study after the CMT, not surprisingly given that the ‘final’ questionnaires assessed constructs (e.g., emotional eating) that may have cued participants to study aims.

**Table S4.1. Awareness Check Statistics**

|  | No | Yes  (During CMT) | Yes  (After  CMT) | Yes  (Indeterminate time) |
| --- | --- | --- | --- | --- |
| Did the participant figure out that they were not playing against real participants? (In other words, did they realize that they were playing against a computer, or think they were playing against confederates instructed on how to behave?) | 25.0% | 40.0% | 10.0% | 25.0% |
| Did the participant figure out that the amount of milkshake they were drinking during each trial was being measured? | 45.0% | 5.0% | 30.0% | 20.0% |
| Did the participant figure out that the aim of the CMT was to examine how exclusion vs. inclusion (or negative vs. neutral mood) affected milkshake intake? | 45.0% | 5.0% | 40.0% | 10.0% |

Abbreviations: CMT = Cyberball-Milkshake Task

# Exploratory Analyses

## Methods

### Interviews and Questionnaires

The ‘final’ questionnaires also included the Emotional Eating Scale (EES; Arnow, Kenardy, & Agras, 1995) and the Rejection Sensitivity Questionnaire (RSQ; Downey & Feldman, 1996).

The EES includes three subscales (Anger/Frustration, 11 items; Anxiety, 9 items; Depression, 5 items). EES items ask whether the respondent feels an urge to eat in response to specific emotions (e.g., “irritated” for Anger/Frustration, “worried” for Anxiety, or “sad” for Depression). Our response options included “A Desire Not To Eat (i.e. Less of a Desire to Eat than Usual)” (=-1), “No (Effect On) Desire to Eat” (=0), “A Small Desire to Eat” (=1), “A Moderate Desire to Eat” (=2), “A Strong Urge to Eat” (=3), and “An Overwhelming Urge to Eat’ (=4), with the first two of these options representing modifications to the original response scale (Arnow et al., 1995), which instead included a “No Desire to Eat” option. In our sample, α = 0.87, 0.85, and 0.77 for the EES Anger/Frustration, Anxiety, and Depression subscales, respectively.

The RSQ presents 18 hypothetical situations with the potential for rejection (e.g., “You ask a friend to do you a big favor.”). The respondent is first asked to indicate their concern about the outcome of the situation (e.g., “How concerned or anxious would you be over whether or not your friend would want to help you out?"), on a 6-point response scale ranging from “Very Unconcerned” (=1) to “Very Concerned” (=6), and then asked to indicate their expectation that the outcome would be one of acceptance (e.g., "I would expect that he/she would willingly agree to help me out."), on a 6-point scale ranging from “Very Unlikely” (=1) to “Very Likely” (=6). The Total Rejection Sensitivity score was calculated as described in Downey & Feldman (1996) by averaging the Rejection Sensitivity scores across situations, with the Rejection Sensitivity score for a given situation calculated as the product of the concern response and (7 – likelihood response). In our sample, α = 0.88 for the situation-specific Rejection Sensitivity scores of the RSQ.

### Data Analysis

#### Exploratory Analyses

We examined Pearson correlations between RSQ Total Rejection Sensitivity scores and difference scores (i.e., “Exclusion” – “Inclusion”) for state psychological variables demonstrating large effect sizes between conditions in planned analyses.

We also examined the effects of Cyberball condition on milkshake intake using CMT trials 2-7 only. (The choice to include these trials was based on examination of milkshake intake values, which were higher on the first trial and lower on the last 5 trials; see Figure 2.) Model 1a examined the effect of exclusion (vs. inclusion) in the sample overall, and Models 2a, 3, 4, and 5 examined how the effect of exclusion (vs. inclusion) differed based on scores on various emotional eating scales. In all models, the outcome variable was milkshake intake (in g), centered around the participant-specific mean, and all models included a random intercept at the participant-level. Predictors in Model 1a were trial-level variables that might influence milkshake intake, including: indicators for the chocolate images that preceded milkshake intake; Trial (i.e., trial number); and an indicator for Condition (exclusion = 1; inclusion = 0). (We used AIC to compare linear, quadratic, and cubic parameterizations for Trial, which indicated that the linear model fit best.) Predictors in Model 2a included all Model 1 predictors, as well as a main effect for DEBQ Emotional Eating and its interaction with the indicator for Condition. Models 3, 4, and 5 were analogous to Model 2a, except DEBQ Emotional Eating was replaced by EES Anger/Frustration, EES Anxiety, and EES Depression, respectively.

## Results

### Sample

Table S5.1 presents descriptive statistics for the EES subscales and RSQ. Notably, some individuals (particularly in the Low Self-Reported Emotional Eating group) endorsed eating less in response to specific emotions.

### Effects of Cyberball Exclusion on Self-Report

RSQ Total Rejection Sensitivity scores were positively correlated with difference scores (i.e., “Exclusion” minus “Inclusion) for Intensity of Ostracism (*r* = 0.42, 95% CI [-0.03, 0.73]), Need Threat Belongingness (*r* = 0.31, 95% CI [-0.15, 0.66]), and Need Threat Socially-Oriented Self-Esteem (*r* = 0.46, 95% CI [0.02, 0.75]), although the confidence intervals for the correlations with Intensity of Ostracism and Need Threat Belongingness included negative values.

### Effects of Cyberball Exclusion on Milkshake Intake

Tables S5.2.a and S5.2.b present results for exploratory linear mixed effects models. In all models, milkshake intake decreased linearly across trials. In Model 1a, the main effect of Condition was -3.49 (95% CI [-5.98, -0.99]), which is in the direction of reduced milkshake intake after exclusion (vs. inclusion). In Model 2a, the interaction between Condition and DEBQ Emotional Eating was 0.26 (95% CI [-1.97, 2.50]); the estimate was in the expected direction (i.e., positive), although the confidence interval included negative values. In Model 3, the interaction between Condition and EES Anger/Frustration was 0.03 (95% CI [-0.25, 0.32]); the estimate was effectively zero and thus not in the expected direction (i.e., positive), although the confidence interval does include positive values. In Model 4, the interaction between Condition and EES Anxiety was 0.33 (95% CI [0.02, 0.64]); the estimate was in the expected direction (i.e., positive), suggesting a more positive change in milkshake intake for Exclusion (vs. Inclusion) for individuals who report a stronger urge to eat in response to anxiety. In Model 5, the interaction between Condition and EES Depression was 0.27 (95% CI [-0.19, 0.73]); the estimate was in the expected direction (i.e., positive), although the confidence interval included negative values.

**Table S5.1. Additional Information for Participants^a^**

|  | Low Self-Reported  Emotional Eating^b^  (*n* = 9) | | | High Self-Reported  Emotional Eating^b^  (*n* = 11) | | |
| --- | --- | --- | --- | --- | --- | --- |
| EES Anger/Frustration | -2.2 | (2.4) | [-7, 0] | 7.8 | (9.7) | [-5, 26] |
| EES Anxiety | -1.1 | (5.2) | [-4, 12] | 5.5 | (8.5) | [-3, 21] |
| EES Depression | 3.6 | (5.0) | [-3, 12] | 8.4 | (4.8) | [0, 14] |
| RSQ Total Rejection Sensitivity Score | 7.9 | (3.1) | [1.7, 12.2] | 8.3 | (4.6) | [3.1, 20.3] |

Abbreviations: EES = (modified) Emotional Eating Scale; RSQ = Rejection Sensitivity Questionnaire

^a^ For continuous variables, statistics include mean (standard deviation) [range].

^b^ Participants had low or high average responses to a subset of modified items from the DEBQ Emotional Eating scale at screening.

**Table S5.2.a. Model Fitting Results for Cyberball-Milkshake Task (Trials 2-7)^a^**

|  | Model 1a^b^ | | Model 2a^c^ | |
| --- | --- | --- | --- | --- |
|  | Estimate | 95% CI | Estimate | 95% CI |
| Intercept | -2.62 | [-7.70, 2.45] | -2.60 | [-8.91, 3.71] |
| Chocolate Image: FP0289 | 0.44 | [-5.81, 6.69] | 0.28 | [-6.10, 6.66] |
| Chocolate Image: FP0675 | 4.05 | [-1.64, 9.74] | 4.06 | [-1.71, 9.83] |
| Chocolate Image: FP0703 | 2.47 | [-2.94, 7.88] | 2.42 | [-3.06, 7.90] |
| Chocolate Image: FP0713 | 3.09 | [-3.10, 9.27] | 2.97 | [-3.34, 9.27] |
| Chocolate Image: FP0083 | 1.71 | [-4.26, 7.68] | 1.64 | [-4.40, 7.68] |
| Chocolate Image: FP0878 | -0.32 | [-6.39, 5.74] | -0.31 | [-6.44, 5.81] |
| Chocolate Image: FP0879 | 2.30 | [-4.19, 8.79] | 2.30 | [-4.25, 8.85] |
| Chocolate Image: IAPS7330 | 4.68 | [-1.85, 11.22] | 4.56 | [-2.09, 11.21] |
| Chocolate Image: IAPS7340 | -0.49 | [-6.86, 5.88] | -0.58 | [-7.03, 5.87] |
| Trial | -1.24 | [-1.95, -0.52] | -1.25 | [-1.98, -0.52] |
| Condition: Exclusion | -3.49 | [-5.98, -0.99] | -4.19 | [-10.46, 2.09] |
| DEBQ EE | -- | -- | 0.00 | [-1.67, 1.67] |
| DEBQ EE x Condition: Exclusion | -- | -- | 0.26 | [-1.97, 2.50] |

Abbreviations: CI = Confidence interval; DEBQ EE = Dutch Eating Behaviour Questionnaire Emotional Eating; FP = FoodPics; IAPS = International Affective Picture System; SE = Standard error

^a^ Outcome variable is milkshake intake in grams, centered based on subject-specific mean milkshake intake.

^b^ Model 1a predictors include Chocolate Image (treated as a dummy coded categorical variable, with FP0167 as the reference category), Trial (with Trial treated as a continuous variables and centered around 6.5), and Condition (treated as dummy coded categorical variable, with Inclusion as the reference category).

^c^ Model 2a predictors include all Model 1 predictors, DEBQ EE (treated as a continuous variable), and DEBQ EE x Condition.

**Table S5.2.b. Model Fitting Results for Cyberball-Milkshake Task (Trials 2-7) – Continued^a^**

|  | Model 3^b^ | | Model 4^b^ | | Model 5^b^ | |
| --- | --- | --- | --- | --- | --- | --- |
|  | Estimate | 95% CI | Estimate | 95% CI | Estimate | 95% CI |
| Intercept | -2.57 | [-7.74, 2.60] | -2.38 | [-7.40, 2.64] | -2.18 | [-7.52, 3.16] |
| Chocolate Image: FP0289 | 0.44 | [-5.88, 6.75] | 0.26 | [-5.95, 6.46] | 0.51 | [-5.77, 6.79] |
| Chocolate Image: FP0675 | 4.05 | [-1.69, 9.79] | 4.13 | [-1.49, 9.75] | 4.43 | [-1.32, 10.17] |
| Chocolate Image: FP0703 | 2.46 | [-3.02, 7.93] | 2.31 | [-3.05, 7.66] | 2.64 | [-2.80, 8.08] |
| Chocolate Image: FP0713 | 3.03 | [-3.23, 9.29] | 2.62 | [-3.50, 8.74] | 3.11 | [-3.10, 9.31] |
| Chocolate Image: FP0083 | 1.68 | [-4.36, 7.72] | 2.03 | [-3.87, 7.94] | 1.85 | [-4.14, 7.85] |
| Chocolate Image: FP0878 | -0.30 | [-6.44, 5.83] | -0.37 | [-6.37, 5.62] | 0.01 | [-6.10, 6.12] |
| Chocolate Image: FP0879 | 2.24 | [-4.36, 8.84] | 2.85 | [-3.59, 9.28] | 2.32 | [-4.19, 8.83] |
| Chocolate Image: IAPS7330 | 4.57 | [-2.10, 11.25] | 4.24 | [-2.23, 10.71] | 4.64 | [-1.93, 11.22] |
| Chocolate Image: IAPS7340 | -0.50 | [-6.94, 5.93] | -0.72 | [-7.05, 5.61] | -0.41 | [-6.81, 6.00] |
| Trial | -1.25 | [-1.97, -0.52] | -1.33 | [-2.04, -0.62] | -1.31 | [-2.03, -0.58] |
| Condition: Exclusion | -3.61 | [-6.33, -0.89] | -4.33 | [-6.92, -1.75] | -5.24 | [-9.13, -1.34] |
| EES AF | -0.02 | [-0.23, 0.20] | -- | -- | -- | -- |
| EES AF x Condition: Exclusion | 0.03 | [-0.25, 0.32] | -- | -- | -- | -- |
| EES AX | -- | -- | -0.16 | [-0.39, 0.08] | -- | -- |
| EES AX x Condition: Exclusion | -- | -- | 0.33 | [0.02, 0.64] | -- | -- |
| EES DP | -- | -- | -- | -- | -0.12 | [-0.46, 0.22] |
| EES DP x Condition: Exclusion | -- | -- | -- | -- | 0.27 | [-0.19, 0.73] |

Abbreviations: CI = Confidence interval; EES AF = (modified) Emotional Eating Scale Anger/Frustration; EES AX = (modified) Emotional Eating Scale Anxiety; EES DP = (modified) Emotional Eating Scale Depression; FP = FoodPics; IAPS = International Affective Picture System; SE = Standard error

^a^ Outcome variable is milkshake intake in grams, centered based on subject-specific mean milkshake intake.

^b^ Models 3, 4, and 5 predictors include Chocolate Image (treated as a dummy coded categorical variable, with FP0167 as the reference category), Trial (with Trial treated as a continuous variables and centered around 6.5), and Condition (treated as dummy coded categorical variable, with Inclusion as the reference category). Model 3 predictors additionally include EES AF (treated as a continuous variable) and EES AF x Condition. Model 4 predictors additionally include EES AX (treated as a continuous variable) and EES AX x Condition. Model 5 predictors additionally include EES DP (treated as a continuous variable) and EES DP x Condition.

# References for Supplementary Material

Arnow, B., Kenardy, J., and Agras, W. S. (1995). The Emotional Eating Scale: The development of a measure to assess coping with negative affect by eating. *International Journal of Eating Disorders* 18, 79–90. doi: 10.1002/1098-108X(199507)18:1<79::AID-EAT2260180109>3.0.CO;2-V.

Blechert, J., Lender, A., Polk, S., Busch, N. A., and Ohla, K. (2019). Food-pics_extended—An image database for experimental research on eating and appetite: Additional images, normative ratings and an updated review. *Frontiers in Psychology* 10, 307. doi: 10.3389/fpsyg.2019.00307.

Blechert, J., Meule, A., Busch, N. A., and Ohla, K. (2014). Food-pics: An image database for experimental research on eating and appetite. *Frontiers in Psychology* 5, 617. doi: 10.3389/fpsyg.2014.00617.

Domoff, S. E., Meers, M. R., Koball, A. M., and Musher-Eizenman, D. R. (2014). The validity of the Dutch Eating Behavior Questionnaire: some critical remarks. *Eating and Weight Disorders - Studies on Anorexia, Bulimia and Obesity* 19, 137–144. doi: 10.1007/s40519-013-0087-y.

Downey, J., and Feldman, S. I. (1996). Implications of rejection sensitivity for intimate relationships. *Journal of Personality and Social Psychology* 70, 1327–1343. doi: 10.1037/0022-3514.70.6.1327.

Lang, P. J., Bradley, M. M., and Cuthbert, B. N. (2008). International affective picture system (IAPS): Affective ratings of pictures and instruction manual. Technical Report A-8. University of Florida: Gainesville, FL.

Nieuwburg, E. G. I. (2015). What to say when they won’t let you play? The effectiveness of cognitive reappraisal on social exclusion. [bachelor's thesis]. [Utrecht (Netherlands)]: Utrecht University.

Sebastian, C. L., Tan, G. C. Y., Roiser, J. P., Viding, E., Dumontheil, I., and Blakemore, S.-J. (2011). Developmental influences on the neural bases of responses to social rejection: Implications of social neuroscience for education. *NeuroImage* 57, 686–694. doi: 10.1016/j.neuroimage.2010.09.063.

Somerville, L. H., Heatherton, T. F., and Kelley, W. M. (2006). Anterior cingulate cortex responds differentially to expectancy violation and social rejection. *Nat Neurosci* 9, 1007–1008. doi: 10.1038/nn1728.

Uskul, A. K., and Over, H. (2017). Culture, social interdependence, and ostracism. *Curr Dir Psychol Sci* 26, 371–376. doi: 10.1177/0963721417699300.
